# True navigation in migrating gulls requires intact olfactory nerves

**DOI:** 10.1038/srep17061

**Published:** 2015-11-24

**Authors:** Martin Wikelski, Elena Arriero, Anna Gagliardo, Richard A. Holland, Markku J. Huttunen, Risto Juvaste, Inge Mueller, Grigori Tertitski, Kasper Thorup, Martin Wild, Markku Alanko, Franz Bairlein, Alexander Cherenkov, Alison Cameron, Reinhard Flatz, Juhani Hannila, Ommo Hüppop, Markku Kangasniemi, Bart Kranstauber, Maija-Liisa Penttinen, Kamran Safi, Vladimir Semashko, Heidi Schmid, Ralf Wistbacka

**Affiliations:** 1 Department of Migration and ImmunoEcology, Max-Planck Institute of Ornithology, Am Obstberg 1, 78315 Radolfzell, Germany; 2 Ornithology, Konstanz University, 78457 Konstanz, Germany; 3 Department of Biology, Via Volta 6, Pisa University, 56126 Pisa, Italy; 4 School of Forest Sciences, Faculty of Science and Forestry, University of Eastern Finland, Joensuu campus, P.O. Box 111, FI-80101 Joensuu, Finland; 5 Karelia University of Applied Sciences, Joensuu, Finland; 6 Institute of Geography, Russian Academy of Sciences, Staromonetnystr. 29, Moscow, 119017, Russia; 7 Center for Macroecology, Evolution and Climate, Natural History Museum of Denmark, University of Copenhagen, Copenhagen 2100, Denmark; 8 Department of Anatomy with Radiology, Faculty of Medical and Health Sciences, University of Auckland. Auckland, New Zealand; 9 Juhannusvuorentie 13, 37600 Valkeakoski, Finland; 10 Institute of Avian Research, An der Vogelwarte 21, 26386 Wilhelmshaven, Germany; 11 Solovetskiy Branch of White Sea Biological Station of Lomonosov Moscow State University, Zaozernaya str. 17-1-6, Solovetskiy, Arkhangelsk district, 164409, Russia; 12 Airport Hohenems, Bahnhofstr. 35, 6923 Lauterach, Austria; 13 Puistotie 4, 67700 Kokkola, Finland; 14 Kalkuntie 1 F 47, 37150 Nokia, Finland; 15 Västäräkintie 7, 80130 Joensuu, Finland; 16 Field Educational Centre “Ecosystem”, Festivalnaya st., 22-8-111, Moscow, Russia; 17 Södra Larsmovägen 139, 68570 Larsmo, Finland

## Abstract

During migratory journeys, birds may become displaced from their normal migratory route. Experimental evidence has shown that adult birds can correct for such displacements and return to their goal. However, the nature of the cues used by migratory birds to perform long distance navigation is still debated. In this experiment we subjected adult lesser black-backed gulls migrating from their Finnish/Russian breeding grounds (from >60°N) to Africa (to < 5°N) to sensory manipulation, to determine the sensory systems required for navigation. We translocated birds westward (1080 km) or eastward (885 km) to simulate natural navigational challenges. When translocated westwards and outside their migratory corridor birds with olfactory nerve section kept a clear directional preference (southerly) but were unable to compensate for the displacement, while intact birds and gulls with the ophthalmic branch of the trigeminal nerve sectioned oriented towards their population-specific migratory corridor. Thus, air-borne olfactory information seems to be important for migrating gulls to navigate successfully in some circumstances.

Migrating birds fly over thousands of kilometres to return to previously visited breeding or non-breeding grounds. Experienced adult birds display the ability to correct for passive displacement from unfamiliar areas^1^, so called true navigation^2^,^3^, almost immediately after release^4^ or in orientation cages at the release site^5^. Current competing theories to explain how the birds locate their position with respect to their final destination propose the predominant use of either magnetic field intensity^6^ or olfactory cues^7^. In fact, some lines of evidence suggest that the intensity of the Earth’s magnetic field may play a role in the navigation of experienced migrants^8^, in which case it may be used as a signal for latitudinal displacement, or as a signpost that a particular latitude has been reached^9^. In contrast, an aerial tracking experiment suggested that an intact olfactory sense might be necessary to correct for experimental displacement in experienced migrating catbirds^10^. The latter work provided the first empirical evidence of the involvement of olfaction in navigation during migration, following on from the demonstration of an odour-based map used in homing in pigeons^11^, and subsequently suggested in wild species, such as swifts^12^, starlings^13^ and shearwaters^14^.

In this study we aimed to investigate the role of different sensory systems in the navigational map of a long distance migrant, the lesser black-backed gull *Larus fuscus fuscus*. To test for the role of olfactory cues we sectioned the olfactory nerves^7^. To test for the role of magnetic cues we sectioned the ophthalmic branch of the trigeminal nerve which has been implicated in the mediation of magnetic information in birds^15^,^16^. Although the same surgical treatment did not impair the navigation of homing pigeons^17^, recently, a trigeminal nerve section affected the ability of Eurasian reed warblers tested in Emlen funnels to compensate for displacement^18^.

To date, the spatial response of migratory birds to experimental displacements performed during migration has relied largely on three techniques: (1) testing changes in the directional tendencies of manipulated birds that display “Zugunruhe”, i.e., migratory restlessness in cages, in which the bird does not move in spatial location but can be exposed to simulated changes in environmental cues, e.g. in the magnetic field^5^, or subjected to sensory manipulation^18^; (2) measuring the initial response of animals after release^4^,^8^,^10^, which does not inform as to whether the bird was successful in reaching its final location; or (3) the recovery of ringed birds^1^, which can be spatially biased due to unequal re-sampling efforts across geographic areas such as Africa or Europe. While advances have been made using these techniques, they do not inform us as to how the animal deals with displacements and with sensory manipulations over the entire course of its migratory journey. To achieve this, GPS location logging combined with satellite data download must be used, and due to the current size limitations, only relatively large-bodied species can be tested.

Finnish and Russian lesser black-backed gulls are long distance migrants, travelling from northern Europe to non-breeding grounds in the greater Lake Victoria region in Africa, a distance of ~7000 km^19^. Gulls do not appear to migrate socially, although they regularly congregate at stopover sites. Gulls generally migrate during the day, but can also fly at night if necessary, such as over water or deserts. During migration, individual black-backed gulls of a shorter-distance migrating sub-species tended to minimize the costs, not the duration of migration, making them slow travellers only achieving distances of ca. 50–100km/day during the migration period^20^. In this study we used experimental long range displacement combined with olfactory or trigeminal nerve section to test which sensory cues are necessary for navigation during migration. Adult birds were captured in southern and central Finland (between 23E 64 N and 30E 61 N) and the White Sea area (Solovki Island) in Russia (36E 65N; Fig. 1). Non-displaced control gulls were tracked from their respective site of capture. Experimental Finnish birds were displaced 1250 km southwest (236°) to Heligoland Island, Germany (8E, 54N; longitudinal displacement of about 1080 km), and experimental Russian birds were displaced 1260 km southeast (139°) to the city of Kazan (Rozhdestveno, 49E, 55N; longitudinal displacement of about 855 km) at the Volga. We expected that birds unable to use sensory information needed for navigation should then continue their migration southward while the birds with their position finding mechanism unaltered should orient towards the population-specific migration corridor^1^,^10^, i.e., approaching the corridor (Fig. 1).

## Results

Non-displaced birds migrated in a narrow front southward towards the western Black Sea, where tracked migrating birds congregated (Fig. 2, Video 1). Thereafter, gulls continued southward towards the eastern Mediterranean and the Nile Delta, where they congregated again and some individuals spent the non-breeding season. As the Nile Delta is likely to be the first important non-breeding season target of the displaced birds, we considered, as the expected direction for the displaced birds, the direction from Heligoland (135°) and from Kazan (208°) to the Nile Delta. The birds that continued their migration southward flew toward the Red Sea from where they migrated in a narrow front towards Lake Victoria and surrounding lakes. We found no significant difference in apparent survival of birds of the experimental groups within the first southward migration (see Methods and Table 1).

The mean vector distributions of the three experimental groups, obtained by averaging the mean direction taken by the birds during their movement, were all significantly different from random according to the One Sample Hotelling test for both Heligoland (C: n = 4, r = 0.78, α = 146° p < 0.01; TriNS: n = 8, r = 0.59, α = 142° p < 0.01; OlfNS: n = 7, r = 0.65, α = 166° p < 0.001) and Kazan releases (C: n = 8, r = 0.70, α = 194° p < 0.001; TriNS: n = 3, r = 0.86, α = 188° p < 0.05; OlfNS: n = 6, r = 0.61, α = 194° p < 0.01) (Fig. 3a,b).

However, from the Heligoland release site, which was far from the migratory route of the non-displaced gulls starting from the Finnish colonies, the anosmic birds oriented in a different direction from both control and trigeminal sectioned birds (Two Sample Hotelling test: OlfNS vs C, F = 6.39 p < 0.05; OlfNS vs TriNS, F = 4.58 p < 0.05). On the contrary, no difference emerged between the directional distributions of control and the trigeminal sectioned birds (Two Sample Hotelling test: C vs TriNS, F = 2.30, p > 0.1) (Fig. 3). Interestingly, the 95% confidence limits of the C (113°–161°) and TriNS (123°–165°) mean vector distributions included the expected direction (135°) towards the first important target area (the Nile Delta, see above), while this direction was not included in the OlfNS distribution confidence limits (147°–184°).

To test whether the African coast might have constituted a leading linear landmark funneling the orientation of the the birds towards the Nile Delta, we restricted the analysis to the part of the tracks ranging from the release site until the first GPS location recorded on the African coast and we obtained comparable results, both in the orientation distributions (One sample Hotelling test: C, r = 0.78, α = 147° p < 0.01, 95% confidence limits 085°–172°; TriNS, r = 0.60, α = 129° p < 0.001, 95% c. l. 102°–162°; OlfNS r = 0.66, α = 164° p < 0.001, 95% c. l. 147°–178°), and the between group comparisons (Two Sample Hotelling test: OlfNS vs C, F = 6.60 p < 0.05; OlfNS vs TriNS, F = 6.01 p < 0.05; C vs TriNS, F = 3.11 p > 0.05). The results of this analysis suggest that the C and TriNS gulls were already oriented towards the expected direction before reaching the African coast.

With regard to the efficiency of the three groups of gulls in approaching the migratory corridor, for the gulls displaced to Heligoland the Two-Way Repeated Measure ANOVA applied to the distance from the migratory corridor at decreasing latitudes (Fig. 4) did not highlight a significant difference between treatments (C, TriNS, OlfNS F_2,16_ = 2.874 p = 0.086), but revealed a significant difference between latitudes (F_24,308_ = 8.716 p < 0.001) and, more importantly, a significant interaction between treatment and latitude (F_48,308_ = 2.892 p < 0.001). In particular, at 40° latitude the distance from the migratory corridor of the anosmic group (OlfNS, n = 6 because one track interrupted before 40° latitude) was significantly greater than that of either the displaced control gulls (Student-Newman-Keuls test p = 0.036; n = 3) or the trigeminal sectioned birds (p = 0.029; n = 6). The statistical difference between the distance from the migratory corridor of the anosmic and the trigeminal sectioned gulls persisted also at lower latitudes (Lat. 39°, p = 0.037; Lat. 38^o^, p = 0.019; Lat. 37°, p = 0.018; Lat. 36°, p = 0.026). This suggests that intact olfactory nerves are required to find the way towards the migration corridor in this displacement.

**Figure 1:**
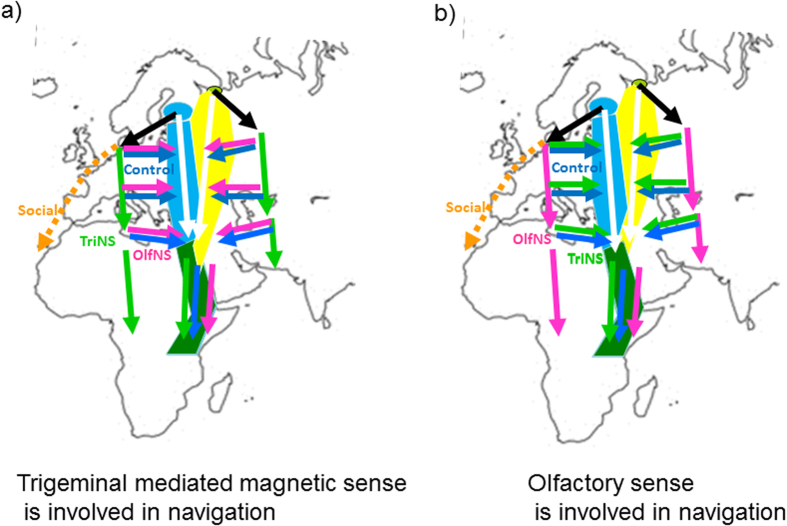
Schematic diagram showing the natural migration corridors of Lesser-black backed gulls from Finland (blue shading) and from the Russian White Sea area (yellow shading) as determined during this experiment, as well as the joint migration corridor of both populations (green shading), and the predicted navigational responses of gulls. Black arrows show the respective translocations, and coloured arrows suggest possible subsequent movement routes of individuals according to the alternative predictions of magnetic and olfactory navigation hypotheses. (**A**) If the trigeminal mediated magnetic sense is involved in navigation, displaced control (blue arrows) and OlfNs birds (red arrows) should correct and return towards the migratory corridor when translocated westward (outside the migratory corridor). TriNs birds (green arrows) should not correct and instead continue their migration southward. (**B**) If the olfactory sense is involved in navigation, displaced control (blue arrows) and TriNs birds (green arrows) should correct whereas OlfNs birds (red arrows) should migrate southward without correction.

**Figure 2:**
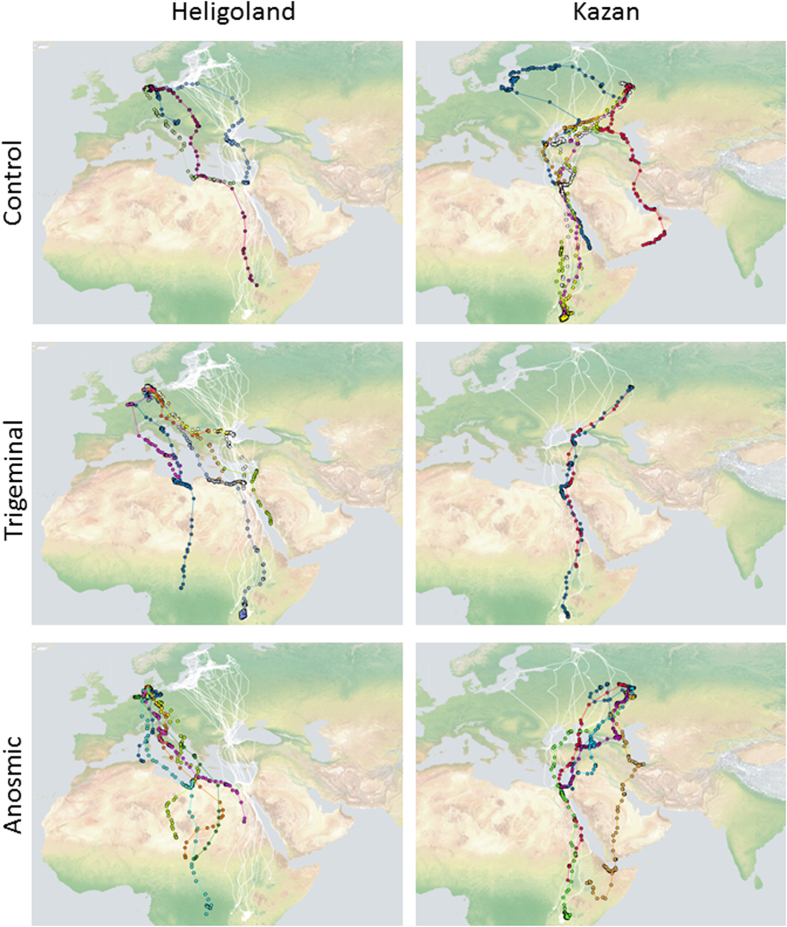
Map of migratory trajectories with white lines showing control birds migrating in the population specific corridor and coloured lines each showing one experimentally translocated bird. Left panel, Finnish birds and birds displaced to Heligoland. Right panel, Russian birds and birds displaced to Kazan. The top panels show Control birds, middle panels trigeminal nerve sectioned birds (TriNS) and bottom panels olfactory nerve sectioned birds (OlfNS, anosmic). Coloured dots indicate GPS locations as reported by the satellite tags. Map drawn on a Natural Earth map (http://www.naturalearthdata.com).

**Figure 3:**
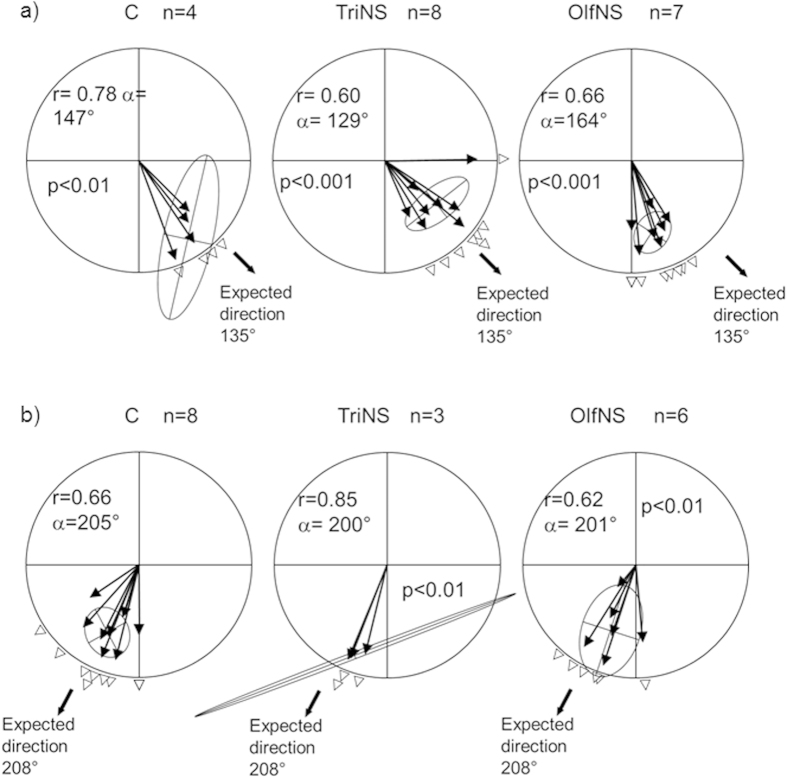
Graph showing the results of the Hotelling Tests for the migration directions for different groups of gulls displaced to (**a**) Heligoland and (**b**) Kazan. The circle indicates the compass rose and the black arrows indicate the directions of individual birds. The length of the arrow shows the directional strength for each bird and, if its pointer falls within the ellipse, indicates that the direction the bird took is within the 95% confidence limit of this group. The arrow outside the compass rose indicates the expected direction. If the expected direction overlaps with the error ellipse, as in C and TriNS birds from Heligoland (**a**), birds correct for their displacement. If the expected direction does not overlap with the error ellipse, as in OlfNS birds from Heligoland (**a**), these birds did not correct for their displacement, but instead continued their migration southward.

**Figure 4:**
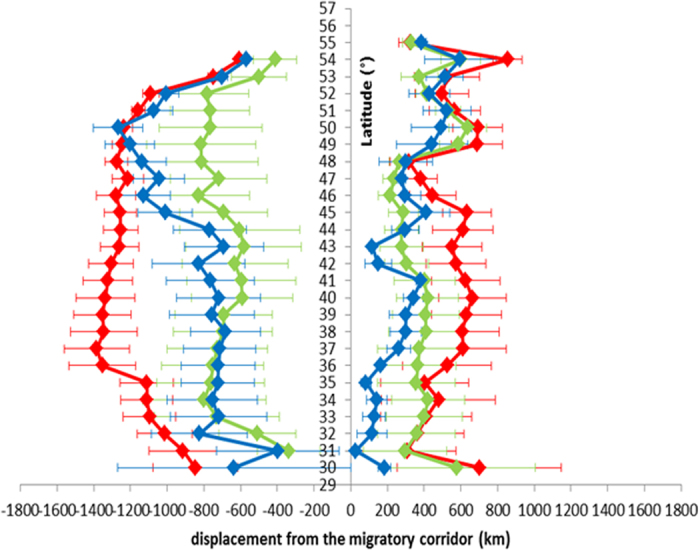
Graph showing the mean distances of translocated birds from the migratory corridor according to latitude. Left panel, Heligoland gulls. Right panel, Kazan birds. Colors as in Fig. 1, displaced control birds (blue tracks), displaced trigeminal nerve sectioned birds (TriNS, green tracks) and displaced olfactory nerve sectioned birds (OlfNS, red tracks). Standard deviation bars are reported.

**Figure 5:**
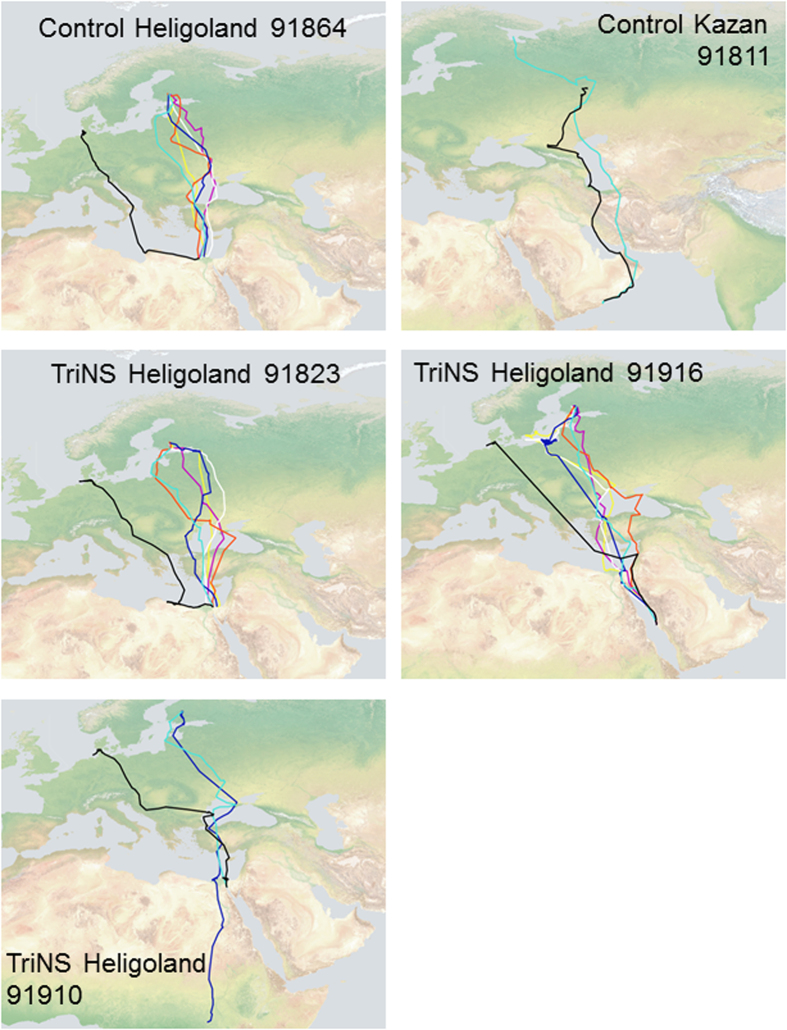
Tracking data showing the migratory trajectories of gulls translocated to Kazan (top right) and Heligoland that could be tracked during repeated return migrations. The black line shows the first southward (fall) migration immediately following translocation, whereas coloured lines show movements in: light blue, spring 2010; dark blue, fall 2010; red, spring 2011; white, fall 2011; violet, spring 2012; yellow, fall 2012. Inset numbers represent the number of the gulls’ satellite tags. Map drawn on a Natural Earth map (http://www.naturalearthdata.com).

The pattern of birds displaced southeast in Russia (C, n = 8; TriNS n = 3, OlfNS n = 6) was different from that in Heligoland. We detected no difference between the mean vector distribution of the three experimental groups (Two Sample Hotelling test, p > 0.25 in all comparisons, Fig. 3). In addition, the expected direction (208°) was included in the 95% confidence limits of all the three experimental groups (95%c.l.: C 176°–215°; TriNS 151°–268°; OlfNS 180°–232°). Consistently, the Two-Way Repeated Measure ANOVA applied to the distances from the migratory corridor (Fig. 4) did not reveal a significant difference, either between treatments (F_2,14_ = 0.852, p = 0.447) or in the interaction between latitude and treatments (F_50,330_ = 0.502, p = 0.998). There was instead a significant difference between latitudes (F_25,330_ = 2.185, p = 0.001). The unimpaired performance of the anosmic birds is possibly due to the fact that the displacement was (unexpectedly) not outside the migratory corridor of non-displaced control birds tagged in Solovki (Figs. 1,2). The tracks of non displaced birds flying over the release area (see Fig. 2, top right panel), and the flight path of a displaced control bird (#91811; Fig. 5) that returned to Kazan before reaching the breeding colony during the subsequent spring suggests that the Solovki birds may have been familiar with the Kazan area. It is worth noting that the spring migratory flights of three TriNS (#91823, #91910, 91916) and one control bird (#91864) displaced to Heligoland were within the population specific migratory corridor far from Heligoland (Fig. 5), suggesting that this release site was unfamiliar to them.

We did not find any differences in the linearity of movement among the experimental groups. The movement tracks of the three groups displaced to Helgoland did not differ in their linearity as shown by a comparison of the mean vector lengths of the tracks (One way ANOVA, p > 0.1; mean r: C 0.78 ± 0.11; TriNS: 0.66 ± 0.11; OlfNS: 0.61 ± 0.16). The same was true for the linearity of the tracks of birds displaced to Kazan (One way ANOVA, p > 0.2; mean r: C 0.73 ± 0.17; TriNS: 0.89 ± 0.05; OlfNS: 0.63 ± 0.24).

## Discussion

Our experiment suggests that intact olfactory nerves are necessary to correct for a longitudinal displacement when displaced outside the migratory corridor to an unfamiliar landscape that did not provide an alternative source of navigational information. Olfactory nerve-sectioned birds were far less likely to orient towards and return to the migratory corridor than both the displaced control and trigeminal nerve-sectioned birds when displaced 1080 km longitudinally west to Heligoland. An impairment of navigational abilities in olfactory nerve-sectioned birds indicates that olfactory cues may play a role in determining longitudinal displacement from the migratory corridor or an intermediate goal area during migration.

The logistics of such a large displacement and multiple groups meant that sham surgery groups, which are a key element of lesion studies, could not be included. This argues for some caution in interpreting our results. However, the different behaviour of the two surgically treated groups in Heligoland argues against non-specific effects of the surgery, trigeminal sectioned birds were still able to correct and return to the migratory corridor. This is further supported by the lack of an effect on successful migration in either group in the eastwards displacement. It should also be noted that sectioning of the olfactory nerves did not affect the birds’ motivation to migrate, their ability to orient southward in the population specific direction, or their ability to reach the latitudes of the wintering areas typical of their population (see Methods and Table 1). Previous experiments on starlings^1^ and white-crowned sparrows^4^ have shown that birds displaced long distances in their first year continue to migrate in a southerly heading (in the northern hemisphere), whereas adults are able to correct for the displacement as they have now developed a navigational map. Thus, the behaviour of anosmic birds in Heligoland is consistent with the removal of the cues responsible for the ability to correct for the displacement and thus reversion to the southerly heading seen by the population. Our data are also consistent with data from anosmic adult catbirds, which oriented southward after an eastward displacement, suggesting a reversion to the population-specific innate migratory direction after removal of navigational cues^10^.

When we displaced gulls southeast within Russia, from Solovki to Kazan, the olfactory nerve-sectioned birds appear to be able to correct for the displacement as proficiently as both the displaced control and the trigeminal nerve-sectioned gulls. Although we cannot be certain that each single bird that was displaced to Kazan had flown to this area before, a few tracks of non-displaced birds and the return track of a displaced control bird indicate that the release site region was not outside the migratory corridor of the Solovki birds. We therefore hypothesize that the Kazan area may have been familiar for the displaced gulls. Alternatively, impaired navigation could also result as a side effect of the nerve section rather than being a specific response to the lack of sensory input. In the current experiment, it seems likely that the impaired navigation was a specific response because (1) only in olfactory nerve sectioned birds was the performance impaired contrasting with the unimpaired performance in trigeminal nerve sectioned birds similar to control birds, and because (2) the navigational performance in olfactory nerve sectioned birds when displaced to a presumed familiar area was unimpaired.

Similar to the gulls translocated to Kazan, anosmic homing pigeons display unaltered navigational performance when released within familiar areas^7^and so intriguingly, this raises the possibility that the birds reverted to different cues specific to the familiar migratory corridor. We urge further investigation of this, but caution is warranted. In the only other experiment testing the role of olfactory cues in migration, anosmic catbirds released on their normal migratory route were oriented in a different direction to controls^10^ and similar to anosmic catbirds that were displaced to an unfamiliar area. Thus, familiarity with the migratory corridor does not always result in the ability to overcome the removal of navigational cues.

The trigeminal nerve-sectioned birds displaced outside the migratory corridor of the non-displaced control population (at Heligoland) were significantly more likely to return to it than olfactory nerve-sectioned birds, indicating that the trigeminal nerve does not play a crucial role in determining longitudinal displacement. This finding is not consistent with that observed by Kishkinev and colleagues in trigeminal sectioned reed warblers displaced and tested in Emlen funnels^18^. However, our and Kishkinev and colleagues’ data are not easily comparable, as they measured the direction of the birds’ migratory restlessness activity for a restricted time window, while we observed the actual movement of the birds in nature during the course of their migratory flights. As the ability to correct for displacement of our TriNS gulls did not emerge immediately after release, the apparent inability of the reed warblers to orient toward the actual goal in the Emlen funnels might not indicate with certainty a total lack of navigational capacity (although it does appear to point to the trigeminal nerve being a necessary component for their initial correction behaviour). Other differences between the two studies included: the species involved; the direction of displacement to an unfamiliar area (west in our study, east in theirs) and linked to this, the nature of the magnetic parameters available in the two studies (intensity north-south in our study, north-east to south-west in their study). The unimpaired navigational abilities of the TriNS gulls are also supported by the tracks of three birds for which we were able to observe, in the years subsequent to the experiment, their successful migratory flights back and forward to their breeding grounds (Fig. 5).

The role of olfactory cues in bird navigation has been mainly studied in homing pigeons^7^ and it has a long and controversial history^21^,^22^,^23^. Nevertheless, a large body of evidence collected over forty years supports a critical and specific role for olfactory information in the navigational map mechanism of homing pigeons^24^. The olfactory map learned at the home loft by associating windborne odours with the wind directions allow the pigeons to determine the direction of displacement on the basis of olfactory cues characterising the release site^25^,^26^. Wallraff and Andreae^27^ provided empirical support to the idea that the atmosphere can provide spatial cues. They showed that ratios of volatile compounds in air sampled over a 400 km diameter area were stable enough to provide spatial information theoretically suitable also for long distance navigation^28^.

The way in which migratory birds might exploit environmental odours for navigation is not known, and the present data only allow theoretical speculations. The ability of experienced adult birds to exploit atmospheric chemical signals to compensate for accidental displacement during their migratory journey was hypothesised by Wallraff^7^. He proposed that birds might learn an olfactory map with a mechanism similar to that described in homing pigeons, both at their breeding/wintering sites and at each stopover site, so to be able to navigate all along their migratory route. Alternatively or in addition, birds might also exploit windborne odour plumes originating from stopover sites within their migratory corridor.

This is one of the first experiments on navigation in migratory birds that documents and takes account of the entire migration route and shows that intact olfactory nerves are essential to allow birds displaced outside of their normal path to return to their population’s migration corridor. Furthermore, its results are consistent with experiments using ringing 50 years earlier^1^ that showed that experienced migratory birds displaced outside their population specific migratory corridor can navigate to their species’ wintering ground and return to their breeding ground the next year. The current experiment provides no support for the trigeminal nerve playing a role in correcting for this mostly longitudinal displacement. Whether magnetic intensity information is conveyed to the brain via the trigeminal system or via the lagena is still matter of debate^29^,^30^,^31^. Our results do not support a role of the Earth’s magnetic field as a cue for navigation. In fact, olfactory deprived gulls with both their trigeminal nerve and lagena intact were unable to orient towards the goal area and approach the population specific migratory corridor, when displaced outside their familiar range.

While there has been great effort in attempting to understand the physiological basis of magnetite-based magnetoreception^15^, outside the laboratory a number of experiments, particularly those reporting tracking data, have not provided support for its role in the navigational map^10^,^14^,^17^,^32^,^33^,^34^,^35^,^36^,^37^. In contrast, the olfactory sense, long neglected in avian species, is increasingly demonstrated to be important in birds’ biology^38^ including navigation.

## Methods

### Experimental birds and surgical manipulations

All experiments and bird handling animal work were conducted according to relevant national and international guidelines. The bird handling and surgical procedures were approved by the MPIO ad hoc IACUC committee. Bird capture and field operations were approved by the administration of the Finnish provinces North Karelia, Ostrobothnia and Pirkanmaa, as well as the Russian authorities of the Ostrov Solovetskiy Archipelago. The translocation experiments were approved by the administration of Pirkanmaa and Ostrov Solovetskiy provincial governments, as well as their veterinary inspection units, both within the EU (Finland to Germany) and within the Russian Federation. A total of 116 adult lesser black-backed gulls (*Larus fuscus fuscus*) were caught in Finland and Russia. All birds were fitted with a ca. 30 gram solar-powered GPS transmitter (Microwave Telemetry Inc., Maryland, USA) using a Teflon harness. The tags were taking 6 GPS location readings per day (if sunlight-induced battery power permitted) with an accuracy of ca. 30 meters. Twenty-four birds were caught using nest traps between May 24 and June 2, 2009 during the breeding season (7 from Kokkola-Uusikaarlepyy, 14 from Tampere and 5 from Eastern Finland, see below), tagged immediately and released. These birds formed the non-displaced control group in Finland. Thirty-six birds were caught in the Tampere rubbish dump, Finland (61° 32′ 35″ N, 23° 58′ 58″ E) between August 8 and 11, 2009. These birds were held in three aviaries for up to six days and divided into three groups: Olfactory nerve sectioned (OlfNS, n = 12), trigeminal nerve sectioned (TriNS, n = 11) and control birds (C, n = 12). Birds were fed locally caught fresh fish daily and had constant access to water. OlfNS and TriNS birds were anesthetized before the surgical treatment. The anesthetic used was ketamine for the birds operated in Finland and chloral hydrate (20% solution, dose 2 ml/kg body weight) for the birds operated in Russia. For nerve sectioning, the surgical procedures were similar to those described for pigeons^16^. In brief, the head was placed in a stereotaxic-like device and held firmly with ear and beak bars. For the birds of the OlfNS group a midline burr hole was drilled through the cancellous bone of the rostral skull so to expose the paired olfactory nerves passing between the bulb and their lateral divergence to their respective epithelia. The nerves were then sectioned bilaterally, their cut ends turned about whenever possible and cyanoacrylate placed between them to prevent re-attachment. For the birds of the TriNS group, the ophthalmic branch of the trigeminal nerve (V1) was sectioned medial to the eye within each left and right orbit following an incision of the orbital fascia at the orbital rim and gentle depression of the globe. A 2–3 mm piece of nerve was removed on each side and the cut nerve ends were sealed with cyanoacrylate. The incisions were then closed with surgical glue. The birds were allowed a minimum of 5 days to recover in groups in outside aviaries with *ad lib* food and water, after which they were fitted with a GPS transmitter and then transported for ca. 6 hours to Heligoland Island (1250km, 236°) by small aircraft (Beechcraft, Bonanza) on 16 August 2009 in individual carrying boxes (600*200*360mm). Once in Heligoland these birds were released immediately at the local airport (54° 11′ 09″ N, 7° 54′ 54″ E). Forty-seven birds were caught in Russia in Solovki (64 59′ 33″ N, 35 40′ 15″ E) between 16–18 August 2009. Twenty birds were used as non-displaced controls and fitted with GPS transmitters, and then released at 6 km south of Solovetskiy (64 59′ 03″ N, 35 43′ 47″ E) on 18–19 August 2009. Twenty-seven birds were retained for displacement to Kazan (1301 km, SE). Again three groups were used: OlfNS (n = 11), TriNS (n = 6) and C (n = 10). We only carried out 6 TriNS surgeries because the optic fibre lamp, critical for the section of the trigeminal nerve, was damaged after two days in the remote camp. These three groups were transported by aircraft (Antonov 26) to Kazan on 19 August 2009 (flight time 5.5 h) and released immediately.

Data relayed from the ARGOS satellites via the CLS (Collecte Localisation Satellites; www.cls.fr) were processed through Movebank (www.movebank.org). Tracks were analysed using Google Earth. Original tracking data are freely available at doi:10.5441/001/1.q986rc29 and at the Movebank DOI (https://www.datarepository.movebank.org/).

### Statistical analysis

To assess potential differences in orientation of the three groups of birds we considered the distributions of the mean vectors computed for each track (again, from the release point up to 30° and 31° Latitude for Heligoland and Kazan birds, respectively) from the direction taken by the bird to move from one fix to the next during its journey. The mean vector distributions were tested for randomness with the One Sample Hotelling test and between group differences in orientation were tested with the Two sample Hotelling test^39^.

Separately for the Finnish and the Russian population, a median migratory corridor to be used as reference for the statistical analysis was calculated. For each degree of latitude from 54° to 30° for the Finnish data set and 55° to 31° for the Russian data set, the median longitude for each non-displaced gull was used to calculate the median longitude for the whole group. To assess the ability of the displaced gulls to correct the displacement we considered the minimal distance from the median migratory corridor of the non-displaced birds, for each latitude degree from 54° to 30° and 55° to 31° for Heligoland and Kazan birds, respectively. Differences in the distances from the migratory corridor of the three groups of gulls were tested with the Two-Way Repeated Measure ANOVA (dependent variable being distance to corridor, one factor being treatment, the other factor being latitude). We calculated the linearity of movement tracks according to methods used in pigeons studies^7^.

### Satellite transmitter discontinuation as a proxy for apparent survival of birds of the three experimental groups

The satellite transmitters used in this experiment (PTT-100 30 gram Solar Argos/GPS PTT from Microwave Inc., Maryland, USA) are reliable technical devices. Similar devices have been used in many field ecology studies over several decades^19^. Nevertheless, they can fail to transmit their information via the ARGOS satellite system for various reasons: (i) The individual carrying the transmitter can die, which generally prevents the solar panel from powering the transmitter; (ii) the transmitter may experience signal interference that is strong enough to block the signal, which is particularly possible around the Mediterranean Sea and in China (CLS, pers. comm.); (iii) the transmitter can fall off the animal, often preventing the solar panel from powering the transmitter, falsely suggesting that the bird is dead when it is actually alive. Here we do not assume a specific probability for the possibilities i-iii, but accept the null model that transmitter discontinuations i–iii occur with similar odds in all groups of birds. Accepting these assumptions, we did not see any difference in apparent survival among the groups of experimental birds during our experimental time of three years (Table 1).

## Additional Information

**How to cite this article**: Wikelski, M. *et al.* True navigation in migrating gulls requires intact olfactory nerves. *Sci. Rep.* **5**, 17061; doi: 10.1038/srep17061 (2015).

## Supplementary Material

Supplementary Information

Supplementary Video 1

## Figures and Tables

**Table 1:** Global categorized location of satellite transmitter discontinuation of birds of the eight treatment groups.

Treatment group	Local within release site (<100 km)	During migration southward (>100 km)	On non-breeding grounds (Egypt or Africa)	% discontinuation of transmitters on non-breeding grounds	Total
Finland control	9	10	15	44%	34
Heligoland control	1	8	3	25%	12
Heligoland OlfNs	1	6	5	42%	12
Heligoland TriNs	0	8	3	27%	11
Russia control	9	3	8	40%	20
Kazan control	3	1	6	60%	10
Kazan OlfNs	5	0	6	54%	11
Kazan TriNs	2	1	3	50%	6
**Total**	**30**	**37**	**49**		116

We did not find any difference between treatment groups (X^2^-Tests, p ≫ 0.05).
