# Supplementary material for: True navigation in migrating gulls requires intact olfactory nerves
Source: Sci Rep. 2015 Nov 24;5:17061. doi: 10.1038/srep17061 (PMC4657012; doi:10.1038/srep17061)
Supplement: Supplementary Information [file srep17061-s1.doc]

True navigation in migrating gulls requires intact olfactory nerves

**Martin Wikelski^1,2,*^, Elena Arriero^1,x^, Anna Gagliardo^3^, Richard Holland^1,y^, Markku J. Huttunen^4^, Risto Juvaste^5,z^, Inge Mueller^1^, Grigori Tertitski^6^, Kasper Thorup^7^, Martin Wild^8^, Markku Alanko^9^, Franz Bairlein^10^, Alexander Cherenkov^11^, Alison Cameron^1,y^, Reinhard Flatz^12^, Juhani Hannila^13^, Ommo Hüppop^10^, Markku Kangasniemi^14^, Bart Kranstauber^1^, Maija-Liisa Penttinen^5,15^, Kamran Safi^1^, Vladimir Semashko^16^, Heidi Schmid^1^ & Ralf Wistbacka^17^**

^1^ Department of Migration and ImmunoEcology, Max-Planck Institute of Ornithology, Am Obstberg 1, 78315 Radolfzell, Germany

^2^Ornithology, Konstanz University, 78457 Konstanz, Germany

^3^Department of Biology, Via Volta 6, Pisa University, 56126 Pisa, Italy

^4^ School of Forest Sciences, Faculty of Science and Forestry, University of Eastern Finland, Joensuu campus, P.O.Box 111, FI-80101 Joensuu, Finland

^5^ Karelia University of Applied Sciences, Joensuu, Finland (Retired)

^6^ Institute of Geography, Russian Academy of Sciences, Staromonetnystr. 29, Moscow, 119017, Russia

^7^ Center for Macroecology, Evolution and Climate, Natural History Museum of Denmark, University of Copenhagen, Copenhagen 2100, Denmark

^8^ Department of Anatomy with Radiology, Faculty of Medical and Health Sciences,

University of Auckland. Auckland, New Zealand

^9^ Juhannusvuorentie 13, 37600 Valkeakoski, Finland

^10^ Institute of Avian Research, An der Vogelwarte 21, 26386 Wilhelmshaven, Germany

^11^ Solovetskiy Branch of White Sea Biological Station of Moscow State University, Zaozernaya str. 17-1-6, Solovetskiy, Arkhangelsk district, 164409, Russia

^12^ Airport Hohenems, Bahnhofstr. 35, 6923 Lauterach, Austria

^13^ 13. Puistotie 4, 67700 Kokkola, Finland

^14^ Kalkuntie 1 F 47, 37150 Nokia, Finland

^15^ Västäräkintie 7, 80130 Joensuu, Finland

^16^ Field Educational Centre “Ecosystem”, Festivalnaya st., 22-8-111, Moscow, Russia

^17^ Södra Larsmovägen 139, 68570 Larsmo, Finland

Current addresses:

^x^ Department of Zoology and Physical Anthropology, University Complutense of Madrid, E-28040 Madrid, Spain

^y^ School of Biological Sciences, Queens University Belfast

^z^ University of Turku, Department of Biology, FI-20014 Turun yliopisto, Finland.

^*^ wikelski@orn.mpg.de

**Supplementary Information**

**Video 1.**

Schematic animation of the movement of individual gulls (pink squares) and winds (white dots) during the southward migration of fall 2009. Degrading red lines show the path of an individual over the past 2 days, pink lines indicate the path of individuals over their entire migratory journey. Please note the translocation of individuals from Finland to Heligoland (SW) and from the Russian White Sea area to Kazan (SE). Please also note the high density of birds in the intermediate goal areas of the SW parts of the Black Sea as well as the Nile Delta of the Mediterranean, as well as the final wintering area for many birds (Lake Victoria). Map made by software freely available through www.movebank.org.
